# Serological and molecular expression of Hepatitis B infection in patients with chronic Hepatitis C from Tunisia, North Africa

**DOI:** 10.1186/1743-422X-7-229

**Published:** 2010-09-15

**Authors:** Samar Ben Halima, Olfa Bahri, Nadia Maamouri, Imed Cheikh, Nissaf Ben Alaya, Amel Sadraoui, Ons Azaiez, Msaddak Azouz, Nabyl Ben Mami, Henda Triki

**Affiliations:** 1 Laboratory of Clinical Virology, Institut Pasteur, Tunis, Tunisia; 2 Departement of Gastroenterology, Hôpital La Rabta, Tunis, Tunisia; 3 Departement of Gastroenterology, Hôpital Bizerte, Tunisia; 4 Laboratory of Epidemiology, Institut Pasteur, Tunis, Tunisia; 5 Departement of Gastroenterology, Hôpital Nabeul, Tunisia

## Abstract

**Background:**

This study reports the prevalence and the viral aspects of HBV infection in HCV-positive patients from Tunisia, a country with intermediate and low endemicity for hepatitis B and C, respectively.

**Results:**

HBV infection was assessed in the serum samples of 361 HCV-positive patients and compared to a group of HCV negative individuals. Serological markers were determined by ELISA tests and HBV DNA by real-time PCR. HBV serological markers were found in 43% and 44% of patients and controls, respectively. However, the serological and molecular expression of HBV infection differed in the two groups: The group of patients included more individuals with ongoing HBV infection, as defined by the presence of detectable HBsAg and or HBV DNA (17% and 12%, respectively). Furthermore, while most of the controls with ongoing HBV infection expressed HBsAg, the majority of HCV and HBV positive patients were HBsAg negative and HBV DNA positive. Genotyping of HCV isolates showed large predominance of subtype 1b as previously reported in Tunisia. Comparison of the replicative status of the two viruses found low HBV viral load in all co-infected patients as compared to patients with single HBV infection. In contrast, high levels of HCV viremia levels were observed in most of cases with no difference between the group of co-infected patients and the group with single HCV infection.

**Conclusions:**

This study adds to the knowledge on the prevalence and the virological presentation of HCV/HBV dual infection, providing data from the North African region. It shows that, given the local epidemiology of the two viruses, co-infected patients are likely to have low replication levels of HBV suggesting a suppressive effect of HCV on HBV. In contrast, high replication levels for HCV were fond in most cases which indicate that the presence of circulating HBV-DNA does not necessarily influence HCV replication.

## Background

Hepatitis C and B viruses (HCV and HBV) are leader causes of chronic liver disease worldwide with 170 and 350 million of individuals infected by these viruses, throughout the world, respectively [1,2]. The two viruses are responsible of multiple liver damages ranging from minor histological disorders to liver cirrhosis and hepatocellular carcinoma (HCC). Severe liver diseases are more frequent when patients are co-infected by the two viruses [3,4]. The interaction between the two viruses in terms of replication activity and the contribution of each virus in the genesis of liver damages remain poorly understood. Several studies found that co-infected patients have lower HBV DNA levels as compared to patients infected with HBV only, suggesting that HCV suppresses HBV replication [5,6], while other studies found no significant difference [7] or even found that HBV suppresses HCV replication [8,9].

Combined HBV/HCV infection is possible because of common modes of viral transmission [10]. It is particularly frequent in areas where the two viruses are endemic and in subjects with high risk of infection through parenteral routes. Depending on the geographic region, less than 1% to 48% of patients with HCV infection were reported to be also positives to hepatitis B surface antigen (HBsAg) [11,12] while 3 to 30% of those with HBV infection were anti-HCV positives [13-15]. Co-infection rates based on the positivity of antibodies to HCV together with HBsAg may also underestimate the true number of patients with dual infection, several studies reported occult HBV infection with detectable HBV DNA and undetectable HBsAg [16].

Tunisia counts among countries with intermediate endemicity for HBV, the published rates of HBsAg positives in blood donors and the general population range from 4 to 7% [17,18]. In contrast, HCV endemicity is low with less than 1% of anti-HCV seropositives [18,19]. Up-to-date, very little was published on HBV/HCV co-infection. Therefore, this case-control study was conducted to assess the prevalence and the virological presentation of HBV infection in 361 Tunisian patients with chronic hepatitis C, in comparison to 361 anti-HCV negative individuals considered as controls.

## Methods

### Studied population

Three-hundred and sixty-one patients' positives to anti-HCV and serum HCV RNA were included together with 361 anti-HCV-negative individuals as control group. The group of HCV-positive patients included 234 females and 127 males (sex ratio M/F = 0.54), aged 18 to 86 years, the median age was 52.0 years. All of them are patients with chronic HCV infection, as defined by persistent positivity of HCV serology and RNA for a minimum of 6 months. They were followed for their anti-HCV positivity in different hepato-gastroenterology departments and sampled for HCV viral load and HCV genotype as part of a pre-treatment investigation. The control group had the same sex ratio and the median age was 51.9 years old (range 18 to 89 years). None of the patients had history of current excessive alcohol intake or of intravenous drugs use. Patients with metabolic and/or autoimmune causes of liver disease were not included. None of the patients and controls was infected by HIV, none was previously vaccinated against hepatitis B.

### Serological and molecular tests

Antibodies to HCV were assessed using a commercial ELISA test from Abbott-Murex (Murex anti-HCV version 4.0). HCV viremia and genotype were assessed by commercial real-time PCR and hybridization tests (Cobas TaqMan Roche and Inno-Lipa, Innogenetics, respectively). Samples with genotype 1 but un-identified subtype were assessed by partial sequencing in the NS5b genomic region using previously described protocol [20]. The presence of HBsAg and antibodies to hepatitis B core and surface antigens (HBcAb and HBsAb) was assessed in all patients and controls using commercial ELISA kits from BIORAD, France (Monolisa HBsAg ULTRA, Monolisa anti- HBc PLUS and Monolisa anti-HBs PLUS). HBV DNA was detected and quantified by Real-time PCR using the commercial test from Roche Diagnostics (COBAS TaqMan HBV test) in all patients and controls positive for HBsAg and those isolated HBcAb.

### Statistical analysis

A descriptive analysis for the data was carried out. The quantitative variables were described by mediane (md) and interquartile range (RI) if the data did not follow a normal distribution and for the categorical variables the percentages were calculated. The chi-square test was used to compare qualitative variables, while T test or ANNOVA and the corresponding non-parametric tests were used to compare the quantitative variables. SPSS version 13.0 was used for statistical analyses.

## Results

HBsAg, HBcAb and HBsAb were detected in 5%, 43% and 17% of HCV positive patients and in 9%, 44% and 25% of HCV negative controls, respectively. HBV DNA was assessed in all HBsAg positive patients and controls, it was also performed most of patients and controls expressing isolated HBcAb (65 out of 74 and 23 out of 34 controls, respectively). The twenty remaining other cases characterized by isolated HBcAb could not be tested because a lack of sufficient quantity of serum (9 patients and 11 controls). According to their status against the different HBV markers, the patients and controls were divided into different groups as shown in Table 1. The group designated "HBV negatives" included all patients and controls who were negative for all HBV markers, thus with no evidence of previous exposure to HBV. The group designated "HBV positives" comprises all individuals with resolutive HBV infection, as defined by the presence of HBcAb and HBsAb; and those with confirmed ongoing HBV infection, characterized by the presence of detectable HBsAg and/or HBV DNA in the serum. The distribution of the studied populations according to these different HBV statuses is shown in Table 1. The status against HBV remained uncertain in the 30 patients and 25 controls expressing isolated HBcAb for whom HBV DNA was negative or could not be assessed. None of the patients and controls had isolated HBsAb.

**Table 1:** HBV serological and molecular markers in HCV positive patients and HCV negative controls

HBV serological status	HCV(+) patients	HCV(-) controls	P value
	N = 361	N = 361	
	No(%)	No(%)	
**All HBV markers negative**	206 (57%)	202 (56%)	0.763
HbsAg(-)/HbcAb(-)/HbsAb(-)			

**HBV positives**			
Resolutive infection			
HbsAg(-)/HbcAb(+)/HbsAb(+)	63 (17%)	92 (25%)	0.008
Ongoing infection			
HbsAg(+)/HbcAb(+)/HbsAb(-/HBV DNA(+)	18 (5%)	33 (9%)	0.029
HbsAg(-)/HbcAb(+)/HbsAb(-)/HBV DNA(+)	44(12%)	9(2.5%)	< 103
**Total**	**62(17%)**	**42(12%)**	**0.03**

HBV negatives counted for 57% and 56% of patients and controls respectively, with no statistically significant difference between the two groups (p > 0.5). The group of HCV-positive patients included less individuals with resolutive HBV infection and more patients with ongoing HBV infection (p < 0.01 and p = < 0.05, respectively). Thus, HBV ongoing infection was found in 62 patients (17%) among which 18 (5%) expressed HBsAg (overt HBV infection) and 44 (12%) were HBsAg negative and HBV DNA positive (occult HBV infection). HBV ongoing infection was found in 42 controls (12%) most of them expressing HBsAg (9%). Thus, the group of HCV positive included much more individuals with occult HBV infection as compared to the control group (p < 0.01).

Table 2 shows HCV genotypes and the demographical characteristics of the HCV-positive patients, divided into 4 sub-groups according to their HBV status: HBV negatives, patients with resolutive HBV infection, HBV/HCV co-infected with overt HBV infection (HBsAg positives) and HBV/HCV co-infected with occult HBV infection (HBsAg negatives). The 30 patients with uncertain HBV status were not included. The mean age was significantly higher in patients with occult HBV infection as compared to HBV negatives (p = < 0.05) and to patients with resolutive infection (p = < 0.05). In contrast, the patients' distribution according to gender was similar in the 4 subgroups. The genotype of infecting HCV viruses could be assessed in 356 out of the 361 patients; the remaining 5 patients had low serum HCV RNA amounts and failed to amplify with the genotyping primers. Subtype 1b was identified in most of patients (85%, 310 out of 361); its frequency was similar in the 4 sub-groups of patients whatever their HBV status was. Subtype 1a and genotypes 2, 3 and 4 were detected in 5% (N = 19), 7% (N = 26), 1% (N = 6) and less than 1% (N = 2) of patients, respectively. Seven patients had mixed infections: 1a and 2 in 4 cases, 1a and 1bin 2 cases and 1a and 4 in one case.

**Table 2:** Demographical characteristics and HCV genotype of HCV positive patients according to their HBV status

	HBV (-)	Resolutive HBV infection	Ongoing HBV infection	Ongoing HBV infection	
			*Overt HBV infection*	*Occult HBV infection*	
	N = 206	N = 63	N = 18	N = 44	P value
	No (%)	No (%)	No (%)	No (%)	
**Age **(Mean = 51.5-SD = 12.1)	50.8	50.0	52.2	56.3	0.011

**Sex ratio **(M/F)	0.56(74/132)	0.61(24/39)	0.50(6/12)	0.33(11/33)	NS

**HCV genotype 1b**	174 (85%)	53 (87%)	16 (83%)	42 (95%)	NS

**Figure 1:**
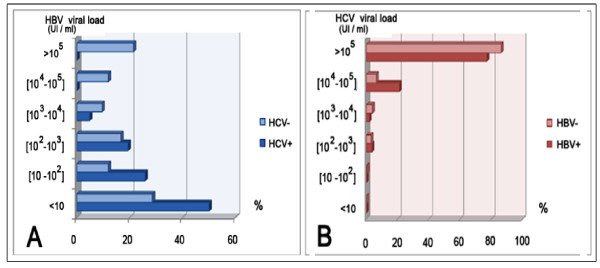
**Comparison of HBV and HCV viral loads in sera of HCV-infected patients and HCV negative-controls**. **Figure 1 (A): **HBV viral load in HBV/HCV infected patients and in HBV infected controls. **Figure 1 (B): **HCV viral load in studied HCV-infected patients.

The viremia levels for HBV and HCV in the studied patients and controls, according to their status against the two viruses, are represented in Figure 1. HBV viremia rates were lower in HCV/HBV co-infected patients as compared to the rates in individuals with single HBV infection (Figure 1A). In contrast, most of the HCV positive-patients had high HCV viral load with similar levels of HCV RNA viremia in HCV/HBV co-infected patients and in those with single HCV infection (Figure 1B).

## Discussion

Due to their wide distribution, HBV and HCV infections count among the most widely studied diseases globally. However, incomplete information is available on co-infected patients in many regions of the world. Dual infection was frequently reported in geographic areas where both infections are highly endemic, such as Southeast Asia. In countries with low endemicity levels for HBV and HCV, such as most parts of Europe and USA, dual infection is mainly found in individuals with high risk for infection with parenterally-transmitted viruses, such as intravenous drug users, hemodialysed patients, patients undergoing organ transplantation and other multi transfused patients [10]. In the North of Africa, few data are available about co-infected patients. Our study is the first reporting HBV infection rates and its virological expression in HCV positive patients from Tunisia, a country with intermediate endemicity for HBV and low endemicity for HCV infection. The rate of HCV positive patients who have also been exposed to HBV infection was equivalent to the one of HCV negative controls (Table 1). These results indicate that the HCV positive patients investigated herein do not have an increased risk of exposure to HBV infection as compared to HCV-negative individuals. Previous studies in Tunisia reported a different geographical distribution of the two infections throughout the country, HBV being more frequent in the southern regions of the country while HCV is more endemic in the north-west [18,20]. It was suggested that the two infections are probably transmitted independently, through different routes of transmission within the community and the results of the present work reinforce this hypothesis. Despite the equivalent rates of HBV positives observed among patients and controls, the serological and molecular expression of HBV infection markedly differed in the two groups. Ongoing HBV infection was found in 12% of controls, most of them expressing HBsAg (9%). It was more frequent in HCV-positive patients (17%), only 5% expressed HBsAg while 12% had occult HBV infection with only HBc-Ab and HBV DNA detected in their serum. Occult HBV infection was previously reported in chronic carriers of HCV from other regions of the world, its prevalence ranged from zero to 52% [16]. It was suggested that this dissimilarity among studies might be due to the heterogeneity of study populations and also to the techniques used to detect HBV DNA which may have different sensitivities. A study from Egypt [21], reported 22.5% of occult HBV infection in 71 patients with chronic HCV infection and HBcAb positives. These results are similar to the rates found herein if we consider only patients with HBcAb positivity. However, both studies may underestimate the real proportion of HCV positive patients with occult HBV infection in the region given the fact that HBV DNA was assessed only in patients with isolated HBcAb among which the probability to find HBV DNA positives is the highest [6]. In fact, HBV DNA has also been reported in the serum sample of patients with no serological markers for HBV, patients with detectable anti-HBs [22]. Also, 30 of our patients expressing HBcAb without HBsAg or HBsAb were not classified among those with ongoing or resolutive HBV infection given that the HBV DNA was negative or could not be assessed. However, many authors classify such patients as occult HBV infection given that many of them have HBV DNA in the liver irrespective to HBV DNA in serum [23]. Accordingly, the prevalence of HCV positive patients with occult HBV infection may be higher but this study suggest that at least 12% of HCV positive patients in Tunisia have an occult HBV infection without detectable HBsAg; this is a significant rate that should be taken into account as part of the treatment and the follow-up of these patients. A lower response to interferon therapy in patients with HCV and occult HBV infection was reported [24-26].

Genotyping of HCV isolates showed that most of patients were infected with subtype 1b whatever was their HBV status (Table 2). The large predominance of subtype 1b was already reported in Tunisia [20,27] and this is another study confirming these results. The prevalence of subtype 1b found herein (85%) is also within the range of the previously reported ones (79% to 88%). A more frequent occult HBV infection was reported in patients infected with subtype 1b, as compared to the other HCV genotypes with high replication levels for HCV and low rates for HBV [28]. In this context, an in vitro study have also demonstrated that the suppression of HBV enhancer 1 by HCV core protein from genotype 1b was stronger than by HCV core protein of genotypes 3a or 1a [29]. We also looked to the HBV and HCV replication levels in our studied population according to their infectious status with the two viruses. The comparison of HBV viral load between individuals with single HBV infection and HCV/HBV co-infected patients revealed significantly lower viremia levels in the second group than in the first one (Figure 1A). These findings indicate that HCV may dominate HBV replication in the group expressing both viruses suggesting that HCV has a suppressive effect on HBV. On the other hand, HCV RNA viremia levels were high in the majority of the patients weather they had a single HCV infection or were co-infected with both viruses (Figure 1B). This indicates that the presence of circulating HBV-DNA does not necessarily influence HCV replication (Figure 1B). Conflicting data were reported concerning the dominant role of either HBV or HCV in co-infected patients. Some reports suggested that the two viruses have a synergistic effect on liver injury while others indicated reciprocal inhibition [10,30,31]. In terms of virus replication, some authors found that HCV have a suppressive effect on HBV [6,32,33] whereas others attributed the suppressive effect to HBV [34]. It was suggested that the type of interaction may depend on the chronology of contamination with the two viruses: HCV super-infection in previously HBV infected patients, co-infection or HBV infection in HCV positives [30,31]. Among the mechanisms accounting for the suppression of HBV replication in coinfected patients, a direct effect of the HCV core protein as a suppressor for HBV replication was suggested [5,35,36]. However, other results from recent in vitro studies ruled out the possibility of direct interference between the two viruses and suggested that the host immune response to HCV infection inhibits in some way HBV replication in the liver cells or possibly in the lymphoid cells [7,36]. Therefore, at present there is no reliable explanation for the interference that should occur "in vivo" between the two viruses.

## Conclusions

The present work adds to the knowledge on the prevalence and the virological expression of HBV infection in patients with chronic hepatitis C providing data from a region where co-infection with the two viruses is not yet well documented. Co-infection is of clinical relevance; it may lead to more rapid progression towards severe forms of liver disease and can interfere with the response to interferon and antiviral therapy. More in vitro studies are required to understand the viral interference in dually infected patients, to identify treatment protocols and to define specific criteria for the follow up of such patients.

## Abbreviations

**HBV: **Hepatitis B Virus; **HCV: **Hepatitis C Virus; **DNA: **Desoxyribonucleic Acid; **RNA: **Ribonucleic Acid; **HCC: **Hepatocellular Carcinoma; **HBsAg: **Hepatitis B surface Antigen; **anti- HCV: **Hepatitis C Virus antibodies; **HBcAb: **Hepatitis B core antibodies; **HBsAb: **Hepatitis B surface antibodies.

## Competing interests

The authors declare that they have no competing interests.

## Authors' contributions

**SBH, OB **and **HT **participated in the study design, in the data analysis and in drafting and discussing the manuscript. **SBH **and **AS **carried out the molecular tests and participated in data **NM, IC, MA **and** NBM **contributed to identification of the patients included into study, providing clinical and epidemiological data and drafting the manuscript. All authors read and approved the final manuscript.
